# Frequency of intron loss correlates with processed pseudogene abundance: a novel strategy to test the reverse transcriptase model of intron loss

**DOI:** 10.1186/1741-7007-11-23

**Published:** 2013-03-05

**Authors:** Tao Zhu, Deng-Ke Niu

**Affiliations:** 1 Ministry of Education Key Laboratory for Biodiversity Science and Ecological Engineering, College of Life Sciences, Beijing Normal University, Xinjiekouwai Street 19, Beijing 100875, China; 2 Beijing Key Laboratory of Gene Resource and Molecular Development, College of Life Sciences, Beijing Normal University, Xinjiekouwai Street 19, Beijing 100875, China

**Keywords:** Free cytoplasmic ribosomes, genomic deletion model, intron loss, long interspersed element, Muridae, processed pseudogene, reverse transcription

## Abstract

**Background:**

Although intron loss in evolution has been described, the mechanism involved is still unclear. Three models have been proposed, the reverse transcriptase (RT) model, genomic deletion model and double-strand-break repair model. The RT model, also termed mRNA-mediated intron loss, suggests that cDNA molecules reverse transcribed from spliced mRNA recombine with genomic DNA causing intron loss. Many studies have attempted to test this model based on its predictions, such as simultaneous loss of adjacent introns, 3'-side bias of intron loss, and germline expression of intron-lost genes. Evidence either supporting or opposing the model has been reported. The mechanism of intron loss proposed in the RT model shares the process of reverse transcription with the formation of processed pseudogenes. If the RT model is correct, genes that have produced more processed pseudogenes are more likely to undergo intron loss.

**Results:**

In the present study, we observed that the frequency of intron loss is correlated with processed pseudogene abundance by analyzing a new dataset of intron loss obtained in mice and rats. Furthermore, we found that mRNA molecules of intron-lost genes are mostly translated on free cytoplasmic ribosomes, a feature shared by mRNA molecules of the parental genes of processed pseudogenes and long interspersed elements. This feature is likely convenient for intron-lost gene mRNA molecules to be reverse transcribed. Analyses of adjacent intron loss, 3'-side bias of intron loss, and germline expression of intron-lost genes also support the RT model.

**Conclusions:**

Compared with previous evidence, the correlation between the abundance of processed pseudogenes and intron loss frequency more directly supports the RT model of intron loss. Exploring such a correlation is a new strategy to test the RT model in organisms with abundant processed pseudogenes.

## Background

The loss of spliceosomal introns in eukaryotic evolution has been well documented [1-11]. However, the molecular mechanism of intron loss is still a matter of debate. Three models have been proposed. The first is the reverse transcriptase (RT) model, also termed mRNA-mediated intron loss [12,13]. This model assumes that a cDNA molecule reverse transcribed from mature mRNA recombines with the intron-present genomic DNA, resulting in the precise deletion of one or several introns from the genomic DNA. The second model describes simple genomic deletion events by mechanisms such as unequal crossover recombination between alleles, which is more likely to cause inexact loss of introns [14-16]. Recently, non-homologous end joining (NHEJ) repair of double strand breaks has been suggested as a mechanism of precise intron deletion [17].

Although the RT model of intron loss is widely cited, it is still controversial and far from being universally accepted. In the yeast *Saccharomyces cerevisiae*, recombination between a reverse transcript and its chromosomal homologue was detected [18,19]. However, in other eukaryotes, both supporting and opposing evidence has been described for the RT model. First, the model predicts that adjacent introns are more likely to be lost simultaneously. In some studies, adjacent intron losses have been found to be significantly higher in frequency than random losses of individual introns [20-26]. By contrast, other studies did not detect a higher frequency of loss of adjacent introns or observe any adjacent intron loss [27-29]. Second, reverse transcription was initially assumed to be primed from the polyadenine tail of mRNA and transcribed towards the 5' end of mRNA. Occasional dissociation of RT from the mRNA template may result in partial-length cDNA, and recombination of cDNA with genomic DNA could cause intron loss preferentially from the 3' end of genes. This bias has been observed in some studies [20,21,25,30-33], but not in others [23,24,27,34-36]. Studies that did not detect this bias tested a modified version of the RT model, the self-primed RT model [37,38]. In this version, reverse transcription was primed by the polyadenine tail of mRNA, and thus the particular intron loss depended on the specific secondary structure of each mRNA molecule. Unfortunately, this model was not supported by analysis of mRNA secondary structures or the frequency of U-rich segments in mRNA molecules [23,24]. Third, for intron loss to occur and be passed to descendants, the RT model requires germline transcription, whereas other models do not. Studies in *Drosophila *and mammals showed that genes expressed in the germline have a higher frequency of intron loss [24,30]. However, the RT model may not be the only possible explanation for the germline expression of intron-lost (IL) genes. Fourth, highly transcribed genes have more substrates for reverse transcription and thus are expected to lose introns more frequently. Intron loss was found to be biased to highly expressed genes in mammals [30], but not in *Arabidopsis *[27].

In this paper, we describe a new approach to test the RT model. In the RT model, the frequency of intron loss depends on the abundance of reverse transcripts, which are thought to be determined by RT activity and the affinity of mRNA templates for RT. Processed pseudogenes (PPs) are the byproducts of RT encoded by retrotransposons [39]. The activity of RT and the affinity of mRNA templates for RT are therefore also determinants of the abundance of PPs [40-42]. Organisms with higher RT activity are thus expected to have more PPs as well as higher frequencies of intron loss than those with lower RT activity. Limited by the small number of PPs annotated in most sequenced genomes, it is difficult to search for such a correlation across a number of species. Within a genome, genes are not identical. Some genes have unique characteristics (high expression in germline cells, co-expression with RT) that increase the frequency of mRNA reverse transcription compared with other genes [40-43]. If the RT model accurately explains the main mechanism of intron loss observed, it would be expected that intron loss and the formation of PPs would frequently occur in parallel among these genes. That is, genes that have more PPs are more likely to lose their introns. PPs are abundant and better annotated in mammalian genomes [44], and a higher rate of intron loss has been identified in mice and rats compared with other mammals [30]. Taking into account these advantages, we studied the relationship between intron loss frequency and the abundance of PPs in mouse and rat species. Our results strongly support the RT model of intron loss in mammals.

## Results

Among the 16,241 intron-containing orthologs between mice and rats, we identified 148,176 conserved introns and 937 unique intron positions that might refer to possible intron losses or gains. By consulting the orthologous genes in seven outgroup mammal species (Additional file 1), we identified 45 intron losses from 42 IL genes in mice and 86 intron losses from 65 IL genes in rats. In addition, 8,410 mouse-rat ortholog pairs were found to consist of only conserved introns and therefore could be definitely defined as no-intron-lost genes (NIL genes). These genes were used as control sets in further analyses.

### Increased processed pseudogenes increase the frequency of intron loss

In rats, we found that 27.7% IL genes were parental genes of PPs. By contrast, among 8,410 NIL genes, only 7.4% were parental genes of PPs. Fisher's exact test showed that the proportion of parental genes of PPs in IL genes was significantly higher than that of NIL genes (*P *= 8 × 10^-7^, Additional file 2). Among the genes that have generated PPs, we found that IL genes produced more PPs than NIL genes. On average, each IL gene produced 0.65 PPs whereas each NIL gene only had 0.19 PPs (Mann-Whitney *U *test, *P *= 3 × 10^-10^).

Also in rats, we found that the parental genes of PPs had a higher rate of intron loss than other genes. On average, each parental gene of PPs lost 0.041 introns whereas each non-parental gene lost only 0.008 introns (Mann-Whitney *U *test, *P *= 6 × 10^-10^). These results showed that intron loss frequency in rats is positively correlated with the abundance of PPs.

**Figure 1:**
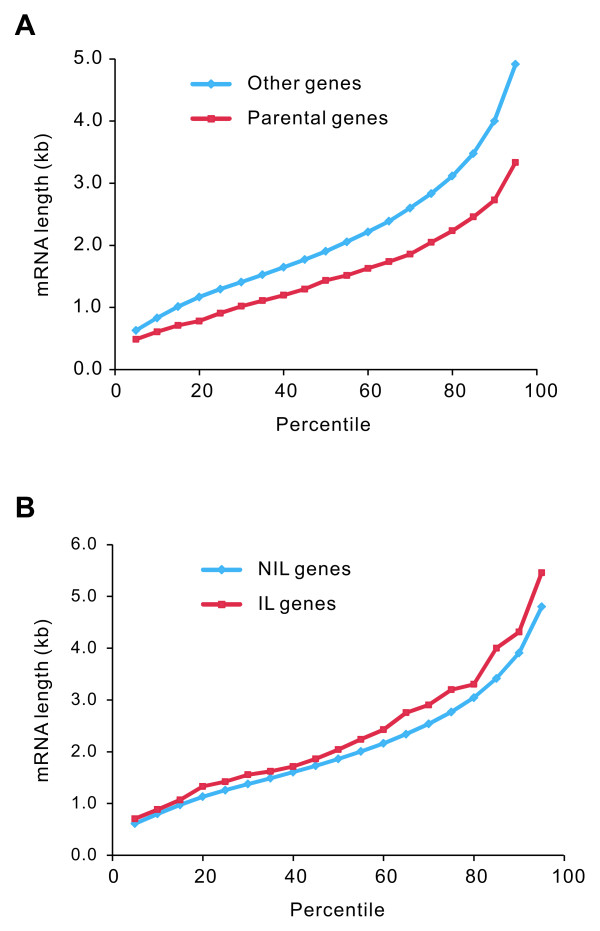
**The relationship between intron loss and processed pseudogene formation was not artifact**. The 5th to 95th percentiles of the data are presented. **(A) **The mRNAs of parental genes of processed pseudogenes were significantly shorter than other genes (*n *= 637, median length = 1.434 kb versus *n *= 7,838, median length = 1.903 kb; Mann-Whitney *U *test, *P *< 10^-16^). **(B) **The mRNAs of intron-lost genes were slightly longer than no-intron-lost genes (*n *= 65, median length = 2.039 kb versus *n *= 8,410, median length = 1.860 kb; Mann-Whitney *U *test, *P *= 0.15). IL: intron-lost; NIL: no-intron-lost.

The PPs produced by genes with short mRNAs are more likely to be detected [42] and genes with fewer introns have relatively shorter mRNAs, so the relationship between intron loss and PP formation might be an artifact. We consistently found that mRNAs of parental genes of PPs were significantly shorter than those of other genes (Figure 1A). However, the mRNAs of IL genes were not shorter, but slightly longer than other genes (Figure 1B). Therefore, the observation that IL genes are enriched in the parental genes of PPs could not be attributed to the artifact resulting from short mRNA lengths.

If genes with more PPs have more introns, they would be more likely to lose introns just by statistical chance. However, we observed a significant negative correlation between the number of extant introns and the abundance of PPs (Spearman Rho = -0.088, *P *< 10^-7^). Genes with larger numbers of PPs had fewer extant introns.

In mice, we observed results almost identical to those in rats described above (Additional file 2). Previously, Coulombe-Huntington and Majewski [30] noticed that the gene for glyceraldehyde 3-phosphate dehydrogenase, *GAPDH*, had multiple pseudogenes with a high rate of intron loss. Based on this observation, they connected reverse transcription to pseudogene formation and intron loss. Our results demonstrated that their observation is not a coincidence and confirmed their findings statistically.

### Parents of processed pseudogenes and intron-lost genes: both enriched in soluble proteins

The transposition machinery of LINEs also serves the transposition of SINEs, the formation of PPs [39,45] and, possibly, the process of intron loss. In eukaryotic cells, membrane proteins and proteins that will be secreted out of cells are translated on the ribosomes attached on endoplasmic reticulum whereas intracellular soluble proteins are translated on free cytoplasmic ribosomes. Proteins encoded by LINEs (the transposition machinery) are translated on free cytoplasmic ribosomes and bind preferentially to the RNAs from which they were translated [45]. To attach the transposition machinery of LINEs, the RNA molecules of SINEs, parental genes of PPs, and IL genes must be translated on free cytoplasmic ribosomes, the same subcellular location as those of LINEs.

The absence of a signal peptide domain or transmembrane domain can be used as a proxy of protein translation on free cytoplasmic ribosomes [42]. We retrieved the signal peptides and transmembrane domains in mouse-rat orthologs from Ensembl BioMart [46]. Consistent with a previous study in humans [42], we found that proteins of parental genes of PPs in mice and rats have significantly higher percentages of soluble proteins than other proteins (Table 1). Among the 65 rat IL genes, about 93% were found to lack signal peptides or transmembrane domains in their proteins (Table 1). By contrast, < 80% NIL genes lacked signal peptides or transmembrane domains. Pearson's chi-square test showed the difference was statistically significant (*P *< 0.05). Similar results were obtained by comparing the IL genes and NIL genes of mice (Table 1). This significantly higher percentage of soluble proteins revealed a common property between parental genes and IL genes: being translated on free cytoplasmic ribosomes. This property suggests that their mRNA molecules are more likely to attach to the LINE transposition machinery, and may thus account for the association between intron loss frequency and the abundance of PPs described above.

**Table 1:** Percentage of genes with signal peptides or transmembrane domains

	*n*	Absence of signal peptides	*P*	Absence of transmembrane domains	*P*
**Rats**					
IL genes	65	92.3%	0.003	93.8%	6 × 10^-4^
NIL genes	8,410	75.7%		74.6%	
Parental genes	637	93.4%	< 10^-16^	90.1%	< 10^-16^
Other genes	7,838	74.4%		73.6%	
**Mice**					
IL genes	42	95.2%	0.004	92.9%	0.015
NIL genes	8,410	74.6%		75.5%	
Parental genes	1,013	91.3%	< 10^-16^	88.3%	< 10^-16^
Other genes	7,439	72.5%		73.9%	

Absence of a signal peptide or a transmembrane domain indicates mRNA translation on free cytoplasmic ribosomes. Pearson's chi-square test was used to calculate the *P *values. IL: intron-lost; NIL: no-intron-lost genes; Parental genes: parental genes of processed pseudogenes.

Pavlicek *et al*. [42] also found that the abundance of PPs was positively correlated with mRNA stability in human genomes. We did not find data on the stabilities of rat mRNA molecules. With the low coverage data of mRNA decay rate [47] and the small sample of intron loss in mice, we compared the mRNA stability between IL and other genes. No significant differences were observed (Mann-Whitney *U *test, *P *= 0.440). Further analyses using a larger sample size or high coverage data of mRNA decay rate are required to reach a definite conclusion.

### Other evidence for the RT model: preferential loss of adjacent introns, 3'-side introns and introns of germline-expressed genes

**Figure 2:**
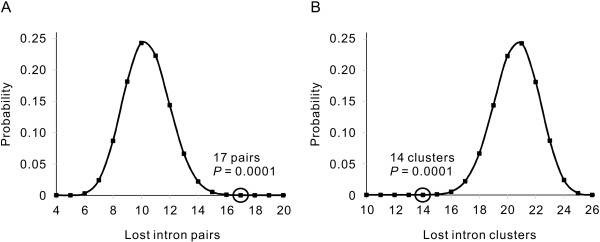
**Adjacent introns tend to be lost together**. The X-axis represents the pairs or clusters of adjacent intron losses and the Y-axis represents their probability of appearance when each intron is lost independently. **(A) **The probability distributions of the loss of adjacent intron pairs. In a case of *n *lost adjacent introns, the adjacent intron pairs were counted as *n *- 1. **(B) **The probability distributions of the loss of intron clusters. The cluster was defined as one or more adjacent introns. For more details of the method, see reference [20]. The observed patterns (marked by circles) have very small probabilities of occurrence via independent loss of each intron.

One prediction of the RT model is that adjacent introns tend to be lost simultaneously, and this has been frequently observed in several species [20-25]. In mammals, however, only a few cases of adjacent intron loss have been reported [30]. In this study, we discovered 10 groups of multiple intron loss in rats, each within a single gene, among which eight groups also had loss of adjacent introns (Table 2). Referring to Roy and Gilbert [20], we calculated the probability distribution of the loss of adjacent introns with the assumption of independent loss of each intron. The obtained probability of 10^-4 ^indicated that adjacent introns tend to be lost simultaneously (Figure 2). In mice, only two genes had lost multiple introns (Table 2).

**Table 2:** Genes losing multiple introns

Ensembl gene ID	Species	Position^a^	Type of loss	Gene symbol
ENSMUSG00000048222	mouse	3,971	adjacent loss	*Mfap1b*
ENSMUSG00000048222	mouse	4,101	adjacent loss	*Mfap1b*
ENSMUSG00000072910	mouse	209	adjacent loss	*Gm16381*
ENSMUSG00000072910	mouse	293	adjacent loss	*Gm16381*
ENSMUSG00000072910	mouse	331	adjacent loss	*Gm16381*
ENSRNOG00000002935	rat	134	adjacent loss	*Ankrd40*
ENSRNOG00000002935	rat	283	adjacent loss	*Ankrd40*
ENSRNOG00000005260	rat	14,955	adjacent loss	*Acp1*
ENSRNOG00000005260	rat	15,061	adjacent loss	*Acp1*
ENSRNOG00000010458	rat	355	adjacent loss	*F1M6Y0_RAT*
ENSRNOG00000010458	rat	564	adjacent loss	*F1M6Y0_RAT*
ENSRNOG00000010458	rat	1,116	solitary loss	*F1M6Y0_RAT*
ENSRNOG00000010989	rat	26,396	solitary loss	*Ipo5*
ENSRNOG00000010989	rat	29,302	solitary loss	*Ipo5*
ENSRNOG00000014048	rat	25,880	adjacent loss	*CYLD_RAT*
ENSRNOG00000014048	rat	26,022	adjacent loss	*CYLD_RAT*
ENSRNOG00000014048	rat	26,145	adjacent loss	*CYLD_RAT*
ENSRNOG00000014048	rat	26,237	adjacent loss	*CYLD_RAT*
ENSRNOG00000014048	rat	26,304	adjacent loss	*CYLD_RAT*
ENSRNOG00000014048	rat	26,437	adjacent loss	*CYLD_RAT*
ENSRNOG00000014048	rat	26,546	adjacent loss	*CYLD_RAT*
ENSRNOG00000020266	rat	2,756	solitary loss	*Eef2*
ENSRNOG00000020266	rat	3,814	solitary loss	*Eef2*
ENSRNOG00000020266	rat	4,435	solitary loss	*Eef2*
ENSRNOG00000025637	rat	444	adjacent loss	*LOC317471*
ENSRNOG00000025637	rat	647	adjacent loss	*LOC317471*
ENSRNOG00000026046	rat	3,972	adjacent loss	*Apex2*
ENSRNOG00000026046	rat	4,237	adjacent loss	*Apex2*
ENSRNOG00000026046	rat	4,384	adjacent loss	*Apex2*
ENSRNOG00000026046	rat	4,454	adjacent loss	*Apex2*
ENSRNOG00000034071	rat	190	adjacent loss	*Chmp4bl1*
ENSRNOG00000034071	rat	368	adjacent loss	*Chmp4bl1*
ENSRNOG00000034071	rat	483	adjacent loss	*Chmp4bl1*
ENSRNOG00000034071	rat	610	adjacent loss	*Chmp4bl1*
ENSRNOG00000043377	rat	7,246	adjacent loss	*Fdps*
ENSRNOG00000043377	rat	7,369	adjacent loss	*Fdps*

^a^The positions of the lost introns in gene sequences, for example, the first row of data represents an intron lost between the 3,971th nucleotide and the 3,972th nucleotide of the *Mfap1b *gene.

**Figure 3:**
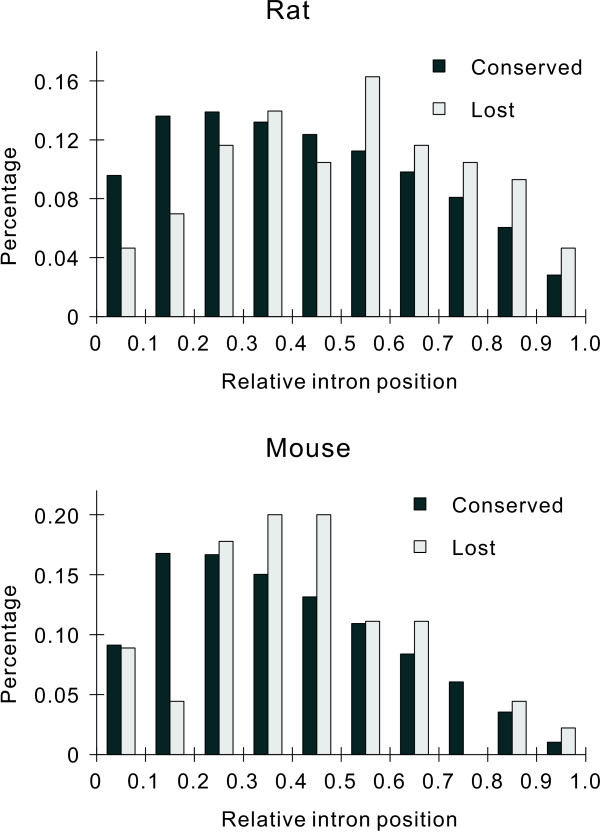
**Relative positions of lost and conserved introns to the 5' end of genes**. In rats, lost introns (*n *= 86) are located closer to the 3' end of genes than conserved introns (*n *= 148,176) (Mann-Whitney *U *test, *P *= 0.001). In mice, the difference is not significant (45 lost introns versus 148,176 conserved introns, *P *= 0.162).

Most copies of LINE are incomplete, indicating that reverse transcription aborts frequently [45]. Because the reverse transcription of mRNA uses the transposition machinery of LINEs [39], it will also frequently abort if the mRNA molecules are longer than LINEs and reverse transcribed from the 3' end. In both mice and rats, we found that mRNA molecules were significantly longer than LINEs (Table 3). Partial-length cDNA molecules are thus expected to be produced frequently. Recombination of these incomplete cDNA molecules with genes causes a preferential loss of introns at the 3' sides of genes. Among the 131 introns lost in mice and rats, 14 introns were 3'-most introns and 86 introns were at the 3' sides of genes. We compared the relative positions of lost and conserved introns, which were defined as the distance of introns to the 5' end of mRNA divided by the whole length of mRNA. In rats, we found that lost introns were more biased to the 3' sides of mRNAs than conserved introns (Figure 3). In mice, the lost introns also appeared to be more biased to the 3' sides of mRNAs than conserved introns, although statistical analysis showed that the difference was not significant (Figure 3). Similar results were obtained when comparing the absolute distance of lost introns and conserved introns to the 3' end of genes (Table 4). It seemed that the RT model accounts less for intron loss in mice compared with rats, thus making results non-significant. Also, the lower intron loss rate (and thus smaller sample size of IL genes) in mice would contribute to this [17].

**Table 3:** Length of mRNA and LINEs in mice and rats

	LINE		mRNA		
		
	*n*	Mean ± SD (kb)	*n*	Mean ± SD(kb)	*P*^a^
Rats	64	1.98 ± 1.52	16,241	2.32 ± 1.73	0.01
Mice	79	1.88 ± 1.37	16,241	3.15 ± 2.35	6 × 10^-12^

Length of LINEs in mice and rats were obtained from Repbase [78]. ^a^Mann-Whitney *U *test was used to calculate the values of *P*. kb: kilobase; LINE: long interspersed elements; SD: standard deviation.

**Table 4:** Distance of introns to the 3' end of genes

	Lost introns		Conserved introns		
		
	*n*	Mean ± SD (kb)	*n*	Mean ± SD (kb)	*P*^a^
Rats	86	1.11 ± 0.80	148,176	2.05 ± 1.99	8 × 10^-9^
Mice	45	2.66 ± 1.97	148,176	2.83 ± 3.14	0.414

Only the length of exonic sequences was calculated. ^a^Mann-Whitney *U *test was used to calculate the values of *P*. kb: kilobase; SD: standard deviation.

In the RT model of intron loss, the mRNA molecules to be reverse transcribed must be expressed in germline cells. We obtained the microarray expression data of mice and rats from BioGPS (Mouse MOE430 and Rat RGU34A Gene Atlas) [48]. In total, 10,291 NIL genes and 57 IL genes had expression data and were thus analyzed. In these datasets, germline cells, like fertilized egg, are absent. We did not use the expression data of reproductive organs (testes and ovary) because they are a mixture of germline cells and supporting somatic cells. Instead, housekeeping genes were used to represent germline-expressed genes. The housekeeping genes were defined as those expressed in all tissues and organs. Genes with values > 200 in the expression data in an organ were defined as expressed in the organ. Consistent with that predicted by the RT model of intron loss, we found that housekeeping genes experienced more frequent intron loss than other genes (Table 5).

**Table 5:** Housekeeping genes are more likely to lose introns

	Number of genes^a^	Housekeeping genes^b^	*P*^c^
**Mice**			
IL genes	39	20.5%	0.012
NIL genes	7,849	8.1%	
**Rats**			
IL genes	18	16.7%	0.066
NIL genes	2,370	5.2%	

^a^Only genes having gene expression data are shown here. Thus, the number of IL genes in this table is smaller than the total number of detected IL genes. ^b^Total number of housekeeping genes: mice, 972; rats, 178. ^c^Fisher's exact test was used to calculate the values of *P*. IL: intron-lost; NIL: no-intron-lost.

### Contribution of the RT model as compared with other models of intron loss

Besides the evidence supporting the RT model of intron loss, we also searched for evidence of genomic deletion and the NHEJ model. First, in our dataset of intron loss, there were seven imprecise intron losses in mice and rats (Additional file 3), accounting for 5.3% of all lost introns. Among these cases of imprecise intron deletion, none had produced any PPs. Second, using the method of Fawcett *et al*. [27], we searched for micro-homology between 5' and 3' splice sites, which might represent NHEJ repair events, for all lost introns. Direct repeats around intron splice sites were found in 12 cases of intron loss in mice and 12 cases in rats. However, such micro-homology also happened at similar frequency around conserved introns (mice, 12 out of 45 versus 29,076 out of 148,176, *P *= 0.32; rats, 12 out of 86 versus 28,914 out of 148,176, *P *= 0.24). Therefore, we found evidence of genomic deletion but no evidence of the NHEJ model of intron loss in mice and rats.

Because we focused only on introns in regions correctly aligned, large insertions or deletions of exon sequences arising during intron loss could not be included in this study. From our result, the conclusion is that both the RT model and genomic deletion model account for some cases of intron loss. It is premature to precisely quantify the relative frequency of intron loss occurring by the mechanisms proposed in the two models. In the future, comparison of very closely-related genomes that could be easily aligned may reveal the relative frequency of exact and inexact intron loss and be helpful to determine whether the RT model is the dominant mechanism of intron loss.

## Discussion

In the RT model of intron loss, recombination between intron-containing genomic DNA and cDNA reverse transcribed from mature mRNA results in the loss of introns from the gene [12,13]. Previous support for the RT model includes the loss of adjacent introns, 3'-side bias of intron loss, 5'-side bias of extant introns, and intron losses biased to genes highly expressed and germline expressed. However, conflicting results have been reported for adjacent intron loss and positional bias of lost introns (see Background). The 5'-side bias of extant introns is less disputable [13,49,50], but alternative explanations have been posited that have not been disproved [49]. Although germline expression of IL genes has not been widely reported [24,30], no conflicting evidence is available. However, we propose an alternative explanation. A large number of studies have revealed an association between transcription and DNA damage including DNA double strand breaks [51-54]. In addition, the NHEJ repair of double strand breaks was recently suggested to cause intron loss [17]. Germline-expressed genes might have a higher frequency of intron loss resulting from the repair of transcription-associated DNA damage. Similarly, the bias of intron loss in highly expressed genes may also be explained by the NHEJ model [17].

In this study, we adopted a new method to test the RT model based on the common process of reverse transcription between intron loss (as proposed by the RT model) and the formation of PPs. IL genes and parental genes of PPs share properties that may facilitate reverse transcription, such as being translated on free cytoplasmic ribosomes. More importantly, we found a positive correlation between the frequency of intron loss and the abundance of PPs. Our results strongly indicate that reverse transcription is a necessary step in intron loss. IL genes in mammals were found to be highly expressed [30]. We also found that the IL genes of mice and rats have significantly higher expression level than NIL genes (Additional file 4). The abundance of PPs is correlated with the expression level of their parental genes [43], especially in rodents whose PPs are relatively young [55,56]. Therefore, the shared feature of high expression between IL genes and parental genes of PPs suggests a common mechanism (that is, reverse transcription). It could be seen that, in mammals, highly expressed genes provide more substrates for reverse transcription, which in consequence leads to both high frequency of intron loss and high abundance of PPs. It should be noted that the correlations of gene expression level with the frequency of intron loss and the abundance of PPs are not necessarily applicable to all species. As gene expression and genome-wide RT activity evolve rapidly, the present gene expression level that can be used in analyses is not necessarily reflecting that at the time of intron loss and PP formation. A previous study showed that the correlation between PP abundance and the expression level of the parental genes of PPs is stronger for young pseudogenes than for old ones [56].

Beside direct recombination of cDNA with genomic DNA, the RT model has another sub-model: recombination or gene conversion of genomic DNA by intronless PPs [22]. If a PP reciprocally recombines with genomic DNA, the intron lost from the functional gene should appear in the PP. If it is gene conversion of genomic DNA by intronless PPs, the exonic sequences flanking lost introns should be more similar to PPs than exonic regions that are unlikely conversed. By searching these evolutionary traces, we attempted to test this sub-model with our dataset of intron loss. Unfortunately, no convincing results were obtained.

There is another possible but less likely explanation for the correlation between intron loss frequency and PP abundance. Highly expressed genes may be more likely to lose introns to reduce metabolic load and the probability of mis-splicing than lowly transcribed genes. Because highly expressed genes have generated more PPs, the intron loss frequency and PP abundance are linked together superficially by high expression level. The metabolic load of introns was previously supposed to be a selective force to intron length reduction in highly expressed genes [57-60]. However, the energetic cost of a long intron in a highly expressed gene was found to be too trivial to act as a selective force for intron loss or intron size reduction in organisms with small effective population sizes like humans and mice [61]. The splicing of each pre-mRNA molecule has a certain probability of error. A highly expressed gene that has a large number of pre-mRNA molecules to be spliced is thus expected to have more mis-spliced products. However, a recent study revealed that the frequency of splicing error is positively correlated with intron length but not with gene expression level, probably because highly expressed genes generally have small introns [62].

Using the abundance of PPs to test the RT model has limitations. Mammalian genomes have an especially high content of PPs and abundance of retrotransposons. In some organisms, transposable elements are subject to constant turnover [63]. If PPs have the same fate as retrotransposons in these organisms, the abundance of PPs would not reflect the affinity of the mRNA molecules of parental genes to RT. As a consequence, the abundance of PPs would not correlate with the frequency of intron loss, even if it occurred by the mechanism proposed by the RT model. In spite of this, we examined whether a correlation between frequency of intron loss and abundance of PPs exists in *Drosophila *and *Arabidopsis thaliana *using the datasets of intron loss previously published [27,64]. None of the IL genes were found to have produced any PPs. Meanwhile, in the NIL genes, a very small proportion produced PPs in *Drosophila *or *Arabidopsis *(0.23% for *Drosophila *and 0.73% for *Arabidopsis*). Fisher's exact tests showed that the differences between IL genes and NIL genes are not significant in either *Drosophila *or *Arabidopsis *(*P *> 0.6 for both cases). Considered just from this result, it seems that the RT model is not the major mechanism of intron loss in either *Drosophila *or *Arabidopsis*. However, previous studies suggested the RT model might be the major mechanism of intron loss in *Drosophila *but not in *Arabidopsis *[21,24,27]. Considering the very low percentage of parental genes of PPs in *Drosophila *and *Arabidopsis*, it is unreliable to reach a conclusion on the RT model based on PP analysis.

The RT model, the genomic deletion model and the double-strand-break repair model attempt to describe how intron loss occurs. In evolution, the fate of a new mutation may be fixed, eliminated or randomly lost depending on its effect on the fitness of the host organism and the population size of the organism. Except that intron losses are neutral and thus randomly lost or fixed, the mutational models of intron loss cannot fully account for the pattern of intron loss. The inaccuracy of the mutational models to account for the patterns of intron loss in many studies may also be explained by the selective fixation of some special cases of intron loss [65-67]. Mice and rats have more frequent intron loss than humans [30,68] but a similar abundance of PPs [44]. With the assumption that introns are slightly deleterious and thus intron loss is selectively favored, this difference could be explained by the difference in the efficiency of natural selection between humans and rodents.

## Conclusions

Reverse transcription is proposed as the main mechanism of intron loss in many species, and PPs are byproducts of RT. Therefore, if RT accounts for most of the intron-loss events, genes undergoing intron loss would produce more PPs, as observed in our study. This novel evidence of RT-mediated intron loss is consistent with previously proposed evidence, such as adjacent intron loss, 3'-biased intron loss and germline-biased expression of IL genes. We also found that both IL genes and parental genes of PPs are more likely to be translated on free cytoplasmic ribosomes where the LINEs are reverse transcribed. This phenomenon indicates that IL genes and parental genes of PPs are closely linked via their same subcellular translation locations. By contrast, in several imprecise IL genes whose introns were unlikely lost via RT process, no related PPs were found. The correlation between PP abundance and intron loss frequency more directly supports the RT model of intron loss than previously reported evidence. It provides a new strategy to test the model in eukaryotes that are rich in PPs.

## Methods

### Genomes and pseudogenes

We downloaded the genome sequences and gene annotations of mouse (*Mus musculus*), rat (*Rattus norvegicus*) and six outgroup species, the guinea pig (*Cavia porcellus*), European rabbit (*Oryctolagus cuniculus*), human (*Homo sapiens*), cattle (*Bos taurus*), African bush elephant (*Loxodonta africana*) and gray short-tailed opossum (*Monodelphis domestica*) from Ensembl (Release 65) [69], and another outgroup species, the Chinese hamster (*Cricetulus griseus*), from National Center for Biotechnology Information (Build 1.1) [70]. Two rodent species, *C. griseus *and *C. porcellus*, were selected because of their close relatedness with mice and rats whereas the other species were selected as outgroups because of their relatively large genome sizes among sequenced mammal genomes. A larger genome is likely to retain more orthologous genes and introns, which are essential for the identification of intron losses in mice and rats. Genes with obvious annotation errors such as those having coding sequences with non-multiples of three nucleotides or conflicting with their protein sequences were discarded. For genes with alternative splicing isoforms, we retained the longest mRNA for analysis.

Pseudogene annotations for mice and rats were obtained from http://Pseudogene.org (Build 60 for mice and build 50 for rats) [44,71]. Only the pseudogenes classed as 'Processed' were retained for analysis. Because the database used the older version of Ensembl ID for annotation, we used the Ensembl ID History Converter [69] to convert old Ensembl IDs to release 65. The retired IDs were discarded. In total, we obtained 7,745 pseudogenes corresponding to 2,469 parental protein-coding genes in mice and 7,029 pseudogenes corresponding to 2,010 parental protein-coding genes in rats (Additional file 5).

### Identification of orthologs

The best reciprocal BLAST was used to search orthologous proteins between mice and rats, with thresholds of *E *values < 10^-10 ^and identities > 0.35. Where a protein from one species matched multiple proteins from other species (with the same *E *value and identity), the genomic context was used to improve orthology assignment. OrthoCluster 2.0 [72] was used to generate synteny blocks between mouse and rat genomes. Finally, 17,321 one-to-one orthologous proteins were obtained, among which 16,241 intron-containing pairs were retained for further analysis (Additional file 6).

### Detection of unique and shared intron positions

Clustal W 2.0 [73] was used to align mouse-rat orthologous proteins. We repeated this step by using Clustal Omega 1.1 [74] and obtained almost identical results. Using these well-aligned protein segments as fixed markers, we aligned the full-length DNA sequences of the orthologous genes using Clustal W 2.0. Only intron positions that flanked reliable alignments of exon sequences (45 bp exon sequences at each side considered) were retained. The reliable alignments of exon sequences were defined by identities > 0.43, the first quintile of the identities of all orthologous mRNA. All alignments containing unique intron positions were manually checked. In total, 937 unique and 148,119 shared intron positions between mouse and rat orthologs were detected.

### Identification of lost introns using outgroup species

Seven outgroup mammals, *C. griseus*, *C. porcellus*, *O. cuniculus*, *H. sapiens*, *B. taurus*, *L. africana *and *M. domestica *were used to distinguish intron losses from intron gains (Additional file 1). Using the same method mentioned above, we identified and aligned the orthologous genes of the outgroup species with Muridae genes containing unique intron positions.

Considering previous observations that intron losses are frequent but intron gain is rare in rodents [2,30,68], multiple losses from one specific position among the studies species are quite possible, but multiple gains are unlikely. As we only focused on intron losses in this study, the standard parsimony was adopted. That is, if a unique intron between mice and rats was present more frequently in the outgroup species, it was considered an intron loss event in Muridae. In total, we identified 78 putative intron losses from 53 mouse genes and 393 putative intron losses from 131 rat genes.

### Filtration of the intron losses

Of the 184 putative IL genes, 43 were intronless. Besides intron loss, another possibility could be that they were new retrogenes, originating from mature mRNA molecules produced by intron-containing parental genes [75]. In addition to sequence similarity, shared genomic position also reflects recent common ancestry of truly orthologous genes. Thus we examined whether the intronless genes had conserved genomic contexts in mice, rats or the outgroup species. Only three genes were retained in synteny blocks with at least one neighboring gene. Considering the rapid chromosome alterations in rodent evolution [76], genes that do not have conserved genomic contexts are not necessarily retrogenes or pseudogenes. Therefore, some of the other 40 genes may not have been retrogenes or pseudogenes but, for accuracy, they were all discarded.

Furthermore, some putative IL genes may have lost multiple introns and retained a few introns, have been new retrogenes that gained a few introns [77], or have been mis-annotated in the exon-intron structures. Therefore, we examined these putative IL genes and removed 39 genes that were not in conserved synteny blocks.

From the 79 possible retrogenes discarded above, we found 37 genes that had intron-containing paralogs that were retained in synteny blocks with neighboring genes. Among these 37 paralogs, 14 matched better with the orthologous genes than the orthologs identified above when we examined the identities globally. Therefore, the 14 possible retrogenes were replaced by their paralogs in the mouse-rat orthologous gene pairs. Among these 14 gene pairs, three intron losses from two rat genes and 60 conserved introns between mice and rats were identified.

Finally, 45 intron losses from 42 mouse genes, 86 intron losses from 65 rat genes (Additional file 7) and 148,176 conserved introns between mice and rats were retained for analysis. Among the 16,241 mouse-rat orthologous genes, 8,410 pairs contained only conserved introns and had definitely not lost or gained any introns. These genes were used as control sets in further analyses and were conveniently described as NIL genes (Additional file 8).

## Abbreviations

BLAST: Basic Local Alignment Search Tool; bp: base pair; IL: intron-lost; LINE: long interspersed element; NHEJ: non-homologous end joining; NIL: no intron losses; PP: processed pseudogene; RT: reverse transcriptase; SINE: short interspersed element.

## Competing interests

The authors declare that they have no competing interests.

## Authors' contributions

DKN and TZ conceived and designed the analyses. TZ performed the analyses. DKN wrote the manuscript. TZ improved the manuscript. Both authors read and approved the final manuscript.

## Supplementary Material

Additional file 1 **Phylogenetic tree of all mammalian species used in this study**. Click here for file

Additional file 2 **Correlation of intron loss and abundance of processed pseudogenes**. **(A) **Percentage of mouse and rat genes producing processed pseudogenes. **(B) **Comparison of the abundance of processed pseudogenes and mRNA lengths between intron-lost genes and no-intron-lost genes in mice. **(C) **Comparison of lost introns and mRNA lengths between parents of processed pseudogenes and other genes in mice. Click here for file

Additional file 3 **Imprecise intron loss events mediated by genomic deletion**. **(A) **Sequence alignments around seven lost introns. **(B, C) **Another case of intron loss that may be mis-recognized as imprecise deletion. Click here for file

Additional file 4 **Comparison of expression levels between intron-lost genes and no-intron-lost genes**. Click here for file

Additional file 5 **Processed pseudogenes obtained from http://pseudogene.org**. Click here for file

Additional file 6 **16,241 intron-containing orthologous genes between mice and rats**. Click here for file

Additional file 7 **Details for lost introns**. Click here for file

Additional file 8 **List of intron-lost genes and no-intron-lost genes in mice and rats**. Click here for file
