# Supplementary material for: Frequency of intron loss correlates with processed pseudogene abundance: a novel strategy to test the reverse transcriptase model of intron loss
Source: BMC Biol. 2013 Mar 5;11:23. doi: 10.1186/1741-7007-11-23 (PMC3652778; doi:10.1186/1741-7007-11-23)
Supplement: Additional file 1 — Phylogenetic tree of all mammalian species used in this study. [file 1741-7007-11-23-S1.DOC]

Additional file 1. Phylogenetic tree of all mammalian species used in this study. It was obtained from the NCBI Taxonomy database and recent studies on the phylogeny of mammals , and is not scaled according to phylogenetic distances.

1. Murphy WJ, Pringle TH, Crider TA, Springer MS, Miller W: **Using genomic data to unravel the root of the placental mammal phylogeny.** *Genome Research* 2007, **17:**413-421.

2. Prasad AB, Allard MW, Green ED: **Confirming the phylogeny of mammals by use of large comparative sequence data sets.** *Molecular Biology and Evolution* 2008, **25:**1795-1808.
