# Supplementary material for: Frequency of intron loss correlates with processed pseudogene abundance: a novel strategy to test the reverse transcriptase model of intron loss
Source: BMC Biol. 2013 Mar 5;11:23. doi: 10.1186/1741-7007-11-23 (PMC3652778; doi:10.1186/1741-7007-11-23)
Supplement: Additional file 2 — Correlation of intron loss and abundance of processed pseudogenes. (A) Percentage of mouse and rat genes producing processed pseudogenes. (B) Comparison of the abundance of processed pseudogenes and mRNA lengths between intron-lost genes and no-intron-lost genes in mice. (C) Comparison of lost introns and mRNA lengths between parents of processed pseudogenes and other genes in mice. [file 1741-7007-11-23-S2.DOC]

Additional file 2. Correlation of intron loss and abundance of processed pseudogenes

A) Percentage of mouse and rat genes producing processed pseudogenes^a^.

|  | Parental genes | Other genes | Fisher's exact test |
| --- | --- | --- | --- |
| mice |  |  |  |
| IL genes | 10 (23.8%) | 32 (76.2%) | *P =* 0.028 |
| NIL genes | 1,003 (11.9%) | 7,407 (88.1%) |  |
|  |  |  |  |
| rats |  |  |  |
| IL genes | 18 (27.7%) | 47 (72.3%) | *P* = 8 × 10^−7^ |
| NIL genes | 619 (7.4%) | 7,791 (92.6%) |  |

^a^IL genes, intron-lost genes; NIL genes, no-intron-lost genes; Parental genes, parental genes of processed pseudogenes.

B) Comparison of the abundance of processed pseudogenes and mRNA lengths between intron-lost genes and no-intron-lost genes in mice.

|  | *n* | Number of PPs^a^ | *P*^b^ | mRNA lengths (kb)^c^ | *P*^b^ |
| --- | --- | --- | --- | --- | --- |
| mice |  |  |  |  |  |
| IL genes^d^ | 42 | 4.40 ± 3.45 | 0.006 | 4.74 ± 0.49 | 7 × 10^−6^ |
| NIL genes^d^ | 8,410 | 0.33 ± 0.02 |  | 2.82 ± 0.02 |  |

^a^Average number of processed pseudogenes, with standard error.

^b^Mann-Whitney *U* test was used to calculate the *P* values.

^c^Average length of mRNA, with standard error.

^d^IL genes, intron-lost genes. NIL genes, no-intron-lost genes

C) Comparison of lost introns and mRNA lengths between parents of processed pseudogenes and other genes in mice.

|  | *n* | Number of lost introns^a^ | *P*^b^ | mRNA lengths (kb)^c^ | *P*^b^ |
| --- | --- | --- | --- | --- | --- |
| mice |  |  |  |  |  |
| Parental genes^d^ | 1,013 | 0.013 ± 0.0050 | 0.018 | 2.13 ± 0.04 | < 10^−16^ |
| Other genes | 7,439 | 0.004 ± 0.0008 |  | 2.93 ± 0.02 |  |

^a^Average number of lost introns, with standard error.

^b^Mann-Whitney *U* test was used to calculate the *P* values.

^c^Average length of mRNA, with standard error.

^d^Parental genes, parental genes of processed pseudogenes.
