# Supplementary material for: Frequency of intron loss correlates with processed pseudogene abundance: a novel strategy to test the reverse transcriptase model of intron loss
Source: BMC Biol. 2013 Mar 5;11:23. doi: 10.1186/1741-7007-11-23 (PMC3652778; doi:10.1186/1741-7007-11-23)
Supplement: Additional file 3 — Imprecise intron loss events mediated by genomic deletion. (A) Sequence alignments around seven lost introns. (B, C) Another case of intron loss that may be mis-recognized as imprecise deletion. [file 1741-7007-11-23-S3.DOC]

Additional file 3 Imprecise intron loss events mediated by genomic deletion. **A)** Sequence alignments around 7 lost introns. Exonic sequence indel accompanying intron loss were marked in red, and internal regions of introns were replaced by “//” if the introns are too long. **B)** Another case of intron loss that may be mis-recognized as imprecise deletion. If being considered only from the alignment of mouse and rat and the current annotation of the mouse gene, it is a case of imprecise intron loss. However, we found that the sequence, “CTCCCTCCCCAG”, also exists in *Cricetulus griseus* and was annotated as intron sequence. **C)** Alignment of the mouse gene region with its mRNA also showed that the sequence, “CTCCCTCCCCAG”, is not included in exons. Therefore, we concluded that the original annotation is wrong, and so the intron loss in rat is a precise intron deletion.

**A)**

intron-lost gene: mouse, *ENSMUSG00000003226*

ortholog gene: rat, *ENSRNOG00000000796*

intron position in ortholog gene: 32,338 − 32,424

mouse TAAAAAATGTGTTGCTTGTGAGAACCCAGGAAAACAGTT---------------------

rat TACAAAATGTGTTGCTTGTCAGAACCCAGCAAAACAGGCtcctgtctcatcacctgcttc

** **************** ********* *******

mouse ------------------------------------------------------------

rat ttttaaggctggcacttcagatgtaagtaaaacttcaaagagtggatttgatgacatgtt

mouse ------------CAAAGAATGGCATTGTAGTTTGTGCTCGGTGAAAAATGAAGCACATGC

rat tgctaaGAAAGAAGGACAGTGGGATTGTAGTTTGTGCTCAGTGAGAAATGAAGCAAATGC

* * *** **************** **** ********** ****

intron-lost gene: mouse, *ENSMUSG00000024050*

ortholog gene: rat, *ENSRNOG00000006217*

intron position in ortholog gene: 965 − 979

mouse GCAGGTGGCCTGGCTGCCCCGGATCATCCTCGAGGCCCC------------------GCT

rat GCAGGTGGCCTGGCTGCCCCGGATCGTCCTCGAGGCCCAgagagactgcctggcCCAGCT

************************* ************ ***

mouse CCAAGAGAGGACATCGAGAGTGGGGC

rat CCAAGAGAGGACATCGAGGGTGGGGC

****************** *******

intron-lost gene: mouse, *ENSMUSG00000041219*

ortholog gene: rat, *ENSRNOG00000008115*

intron position in ortholog gene: 14,332 − 14,346

mouse CCCAAGGAACCAGCTAAGCACT---CCATCACGGGGGAATGAGAATGGGG---GGGAGAG

rat CCCAGGGAGCCAACTAGATACCACACCGTCACAGGGGGATGAGAATGTGGTGGAGGAGAA

**** *** *** *** ** ** **** **** ********* ** *****

mouse CGCTGGGGCAAGTGGAGCTCCCGGAGAAGATGAGAG------------------CACGTG

rat CTCAGGGGCATGTGAAGCTCCTGAGGAAAATGATGCGCAgtctttaaagcagagCACGTG

* * ****** *** ****** * *** **** ******

mouse CAGTGTGGCGGTCCTTTCGAAACCTCGGCCCCA

rat CAGTGTGACAGTCATTTCAAAACCTCGGGCCCA

******* * *** **** ********* ****

intron-lost gene: mouse, ENSMUSG00000055322

ortholog gene: rat, ENSRNOG00000014182

intron position in ortholog gene: 32,338 − 32,424

mouse GTAGTAGCCGAGAGGCTGTGCAGAGGGGTCTGAATTCAT---------------------

rat GCAGCAGCCGAGAGGCTGTGCAGAGGGGCCTGAATTCATggcagcagcagcagcagcagc

* ** *********************** **********

mouse ------------------------------GGCAACAGCAGCAGCCCCACCCACCTCC

rat agcagcagcagcagcagcagcagcagcagcagcagcagcagcagcCCCACCCACCTCC

*** ***********************

mouse CCGCCAGCAGGAGAGATCCCCACTGCAGAGTCTTGCCCGCAGCAAGC

rat CCGCCAGCAGGAGCGATCCCCACTGCAGAGTCTAGCCTGCAGCAAGC

************************** ********************

intron-lost gene: rat, *ENSRNOG00000000397*

ortholog gene: mouse, *ENSMUSG00000020074*,

intron position in ortholog gene: 45,026 − 45,282

mouse ATGAAGAGTCAGAGGCACTGCAGGAAGACATGCTAGgtttgagtgtatttacatttgg//

rat ATGACGAGCCAGAGTCGCTGCAGGGGGACACGACGCTGG-------------------//

**** *** ***** * ******* **** *

mouse taaaacattttttatattctagGAAACAGATTATTACTTCCAACACCAACAATAAAACAG

rat ----------------------GAAACAGGTTACTACTTCCCACACCAACAGTGAAGCAG

******* *** ******* ********* * ** ***

intron-lost gene: rat, *ENSRNOG00000029621*

ortholog gene: mouse, *ENSMUSG00000023903*

intron position in ortholog gene: 12,722 − 13,396

mouse GGTTCCGCCTCCCCAGCCTCCAGCCATGCCCCCTGACAGgtaagtgtgtgacctatccag

rat --TACCCCCTGCTCAGTCCCTTTTCCTCCCCAG---------------------------

* ** *** * *** * * * * ***

mouse cctccagccatgcccccttgcaggtaagtg//agcaaggcacagggagccccgcctcctg

rat ------------------------------//----------------------------

mouse cttactcccctgtccttcccagCCCCGCCACACCTGTGCCTGACCGCTGTGAGGGCAACT

rat ----------------------CCCCTCCACACCTGTGCCTGATCGATGTAAGGGCAATT

**** **************** ** *** ******* *

intron-lost gene: rat, *ENSRNOG00000042581*

ortholog gene: mouse, *ENSMUSG00000036858*

intron position in ortholog gene: 59 − 4,729

mouse ATGGCTAGGACATGGCTGCTGC-TGCTTCTGGGCGTCAGGTGTCAGGCTCTACCATCAGg

rat ----GCACGTTCTTATTTCTGTGTTTTTAAAGAAATCAAAATTCTTACTCTTGCAG----

* * * * *** * ** * *** ** **** **

mouse tatgtccctcttgcctccctatttggctctcactgagttcatcccctggt//gcattcct

rat --------------------------------------------------//--------

mouse atttctgtgtttttaaagagatcaaaattctcactcttgcagGCATCGCTGGCACCCCCT

rat ------------------------------------------GCATCGCTGGCACCCCCT

******************

mouse TTCCGTCTCTGGCTCCACCCATCACACTGCTGGT

rat TTCCTTCTTTGGCTCCACCCGTCACGCTGCTGGT

**** *** *********** **** ********

**B)**

intron-lost gene: rat, *ENSRNOG00000004515*

ortholog gene: mouse, *ENSMUSG00000020430*

intron position in ortholog gene: 9,395 − 9,462

outgroup ortholog gene: Chinese hamster (*Cricetulus griseus, Cgri*), *gene15934*

rat ATGCACTACGTCATTGCTGCCCGAGCCCTGCGCAAG------------------------

mouse CTGCACTATGTCATCACTGCCCGAGCCCTGCGCAAGgtgagctgaggctcttctggggag

Cgri ATGCACTACGTCATCGCTGCCCGAGCCCTGCGCAAAgtgagctgagactatcctggagct

******* ***** *******************

rat ------------------------------------------------------------

mouse ccctggtcctgccctgtcactccttggctttcatgctgttct----ccCTCCCTCCCCAG

Cgri tgttggacctcctctgtcaccccttggttttcatactgttgtgtcgcctcccacccccag

rat GTCTTCCTGTCCATCAAAGGCATCTACTATCAG

mouse GTCTTCCTGTCCATTAAAGGCATTTATTATCAG

Cgri GTCTTCCTGTCCATCAAAGGCATTTACTATCAG

************** *********** ******

**C)**

mouse: *ENSMUSG00000020430*

related transcription data in mouse: NM_022889.3, mRNA

mouse CTGCACTATGTCATCACTGCCCGAGCCCTGCGCAAGgtgagctgaggctcttctggggag

mRNA CTGCACTATGTCATCACTGCCCGAGCCCTGCGCAAG------------------------

************************************

mouse ccctggtcctgccctgtcactccttggctttcatgctgttctccCTCCCTCCCCAGGTCT

mRNA --------------------------------------------------------GTCT

****

mouse TCCTGTCCATTAAAGGCATTTATTATCAG

mRNA TCCTGTCCATTAAAGGCATTTATTATCAG

*****************************
