# Supplementary material for: Frequency of intron loss correlates with processed pseudogene abundance: a novel strategy to test the reverse transcriptase model of intron loss
Source: BMC Biol. 2013 Mar 5;11:23. doi: 10.1186/1741-7007-11-23 (PMC3652778; doi:10.1186/1741-7007-11-23)
Supplement: Additional file 4 — Comparison of expression levels between intron-lost genes and no-intron-lost genes. [file 1741-7007-11-23-S4.DOC]

Additional file 4. Comparison of expression levels between intron-lost genes and no-intron-lost genes

|  |  | Number of Genes^a^ | Expression Level^b^ | *P^c^* |
| --- | --- | --- | --- | --- |
| Mice |  |  |  |  |
|  | IL Genes^d^ | 39 | 377 | 0.015 |
|  | NIL Genes^d^ | 7,849 | 191 |  |
| Rats |  |  |  |  |
|  | IL Genes^d^ | 18 | 186 | 0.005 |
|  | NIL Genes^d^ | 2,370 | 38 |  |

^a^Only genes having gene expression data are shown here. Thus, the numbers of IL genes in this table are smaller than the total number of detected IL genes.

^b^Median expression level over all organs.

^c^Mann-Whitney *U* test was used to calculate the *P* values.

^d^IL genes, intron-lost genes; NIL genes, no-intron-lost genes.
